# Fat Mass and Obesity‐Associated Protein Contributes to Tumorigenesis and Drug Resistance of Diffuse Large B‐Cell Lymphoma by Suppressing N6‐Methyladenosine Methylation of Myc

**DOI:** 10.1002/kjm2.70158

**Published:** 2026-01-13

**Authors:** Ting‐Ting Lu, Jin‐Hao Chen, Yan‐Fang Wang, Xiao Wu, Hui‐Yun Ni, Qiu‐Rong Zhang

**Affiliations:** ^1^ Department of Hematology The Affiliated Zhangjiagang Hospital of Soochow University Zhangjiagang Jiangsu China

**Keywords:** CRISPR‐Cas9, demethylation, ibrutinib, lymphoma, target

## Abstract

Ibrutinib resistance remains a major obstacle in the treatment of diffuse large B‐cell lymphoma (DLBCL). Fat mass and obesity‐associated protein (FTO) has been implicated in drug resistance through its regulation of N6‐methyladenosine (m6A) modifications; however, whether FTO mediates ibrutinib resistance in DLBCL remains unclear. In this study, we found that FTO expression was significantly upregulated in patient samples and DLBCL cell lines. Functional assays showed that FTO knockout or knockdown suppressed cell proliferation and colony formation, whereas FTO overexpression promoted tumor growth and increased ibrutinib half‐maximal inhibitory concentration. Mechanistically, FTO directly bound to myelocytomatosis oncogene (Myc) mRNA and removed m6A modifications, stabilizing Myc transcripts and enhancing Myc protein expression. Methylated RNA immunoprecipitation–quantitative polymerase chain reaction confirmed that FTO knockout increased m6A enrichment on Myc mRNA, leading to decreased Myc stability. Rescue experiments showed that reintroducing Myc partially restored the proliferative and drug‐resistant phenotype of FTO‐deficient cells. Consistently, in xenograft models, FTO promoted tumor growth and ibrutinib resistance in a manner partly dependent on Myc. Overall, FTO promotes DLBCL progression and ibrutinib resistance by demethylating Myc mRNA and stabilizing its expression. These findings highlight the FTO‐m6A‐Myc axis as a potential therapeutic target for overcoming drug resistance in DLBCL.

## 1 Introduction

Diffuse large B‐cell lymphoma (DLBCL) is an aggressive and genetically heterogeneous lymphoma. It comprises several molecular subtypes, such as the germinal center B‐cell–like and activated B‐cell–like (ABC), which differ in genetic alterations and therapeutic responses. The combination of rituximab, cyclophosphamide, doxorubicin, vincristine, and prednisone (R‐CHOP) remains the standard first‐line therapy for patients with newly diagnosed DLBCL; however, approximately 30%–40% of patients experience relapse or refractory disease [1]. To overcome R‐CHOP failure, therapies targeting the B‐cell receptor (BCR) signaling pathway have been developed [2, 3]. Ibrutinib, a selective Bruton's tyrosine kinase (BTK) inhibitor, exhibits clinical activity primarily in the DLBCL ABC subtype by suppressing BCR signaling [4]. Despite its efficacy, both primary and acquired resistance to ibrutinib occurs frequently, leading to unsatisfactory long‐term outcomes [5]. Therefore, understanding the molecular basis of ibrutinib resistance in DLBCL is key to developing more effective therapeutic strategies.

An increasing number of studies have shown that epigenetics plays a key role in drug resistance in cancer treatment [6]. Beyond classical chromatin modifications, RNA epigenetic regulation, particularly through N6‐methyladenosine (m6A), has recently emerged as an important mechanism controlling mRNA stability and translation. Fat mass and obesity‐associated protein (FTO) is an RNA demethylase that removes m6A modifications from RNA molecules. In cancer treatments, FTO upregulation reduces sensitivity to drugs such as 5‐fluorouracil [7] and doxorubicin [8]. Notably, FTO has also been identified as a prognostic factor in relapsed B‐cell acute lymphoblastic leukemia [9]. However, whether FTO mediates ibrutinib resistance in DLBCL remains unclear. Recent mechanistic studies have shown that acquired ibrutinib resistance in B‐cell lymphomas often involves epigenetic reprogramming of BCR and NF‐κB signaling rather than fixed genetic lesions, supporting a non‐genetic, chromatin‐driven adaptation to BTK inhibition [10]. In parallel, epitranscriptomic m6A programs modulate drug response in DLBCL, exemplified by METTL3 and YTHDF2 control of C1qA that impacts rituximab resistance [11]. Furthermore, in DLBCL, FTO‐mediated m6A demethylation upregulates flotillin 2 (FLOT2) and activates PI3K–Akt–mTOR signaling, linking FTO to aggressive biology in this disease context [12]. These findings provide a strong rationale for investigating whether m6A demethylation by FTO links epitranscriptomic remodeling to oncogenic signaling and BTK inhibitor response in DLBCL, consistent with evidence that FTO can stabilize oncogenic RNAs, including Myc, and influence therapeutic resistance in hematologic malignancies [13, 14]. Nonetheless, the specific target genes and regulatory networks controlled by FTO in DLBCL remain incompletely defined.

Myc, a pro‐oncogene, promotes tumorigenesis and progression by regulating cell cycle, metabolism, and apoptosis‐related genes [15]. Previous studies have revealed that FTO‐mediated m6A demethylation can stabilize Myc mRNA and enhance Myc‐driven oncogenic effects in several cancers [16, 17]. For instance, Xiao et al. demonstrated that FTO inhibition enhanced temozolomide sensitivity by disrupting the MYC–miR‐155/23a–MXI1 feedback circuit in glioma [18]. Yang et al. further showed that in gastric cancer, histone deacetylase 3 represses forkhead box protein A2, thereby sustaining an FTO/m6A/MYC signaling axis that drives tumor development [19]. In colorectal cancer, Yue et al. reported that the AMPKα2‐FTO‐m6A/MYC axis regulates proliferation and drug response [20]. These findings suggest that FTO functions as a critical upstream regulator of MYC and may participate in drug resistance. However, whether FTO regulates Myc via m6A to promote DLBCL tumorigenesis and ibrutinib resistance remains unknown. Therefore, in the present study, we aimed to determine whether FTO regulates ibrutinib resistance in DLBCL by modulating Myc expression through m6A demethylation.

## 2 Materials and Methods

### 2.1 Online Database Analysis

The expression levels of FTO and Myc in DLBCL (*n* = 47) and normal samples (*n* = 337) were obtained from the Gene Expression Profiling Interactive Analysis (GEPIA) database (http://gepiacancer‐pku.cn/index.html). The correlation between FTO and Myc in DLBCL was assessed using the Multi Experiment Matrix (MEM) database (https://biit.cs.ut.ee/mem/index.cgi).

### 2.2 Cell Culture

HMy2.CIR (#CL‐0112; Procell, Wuhan, China) and OCI‐Ly1 cells were cultured in Iscove's Modified Dulbecco's Medium (#12440053; Gibco; Thermo, Waltham, MA, USA) supplemented with 10% or 20% fetal bovine serum (FBS; #10091148; Gibco; Thermo). SU‐DHL‐8 (#CL‐0871; Procell), WSU‐DLCL2 (#CL‐0774; Procell), and U2932 (#CL‐0838; Procell) cells were cultured in RPMI‐1640 (#11875093; Gibco; Thermo) containing 10% FBS and 1% penicillin–streptomycin solution (#PB180120; Procell).

### 2.3 Plasmid Construction, Cell Transfection, and Lentiviral Infection

FTO‐specific shRNAs (FTO‐sh1 and FTO‐sh2), Myc‐specific shRNAs (Myc‐sh1 and Myc‐sh2), and a negative control (sh‐NC) were synthesized by Genepharma (Shanghai, China) and ligated to the pLKO.1‐puro lentiviral vector (#8453; Addgene, Watertown, MA, USA). The full‐length cDNA sequences of FTO and Myc were subcloned into the pLVX‐Puro lentiviral vector (#632164; Clontech, Palo Alto, USA) to construct FTO and Myc overexpression vectors (LV‐FTO and LV‐Myc), with the empty pLVX‐Puro plasmid (LV‐Con) as a negative control. The shRNA oligonucleotide sequences are listed in Table S1. Target cells were infected with lentiviral particles and selected with 2 μg/mL puromycin (#C0351; Beyotime, Shanghai, China) for 3 days.

The single‐guide RNA (sgRNA) sequences targeting FTO (exon 3) and Myc (exon 2) were designed and cloned into the lentiCRISPR v2 vector (#52961; Addgene) to construct lentiCRISPR v2‐sgFTO (sgFTO) and lentiCRISPR v2‐sgMyc (sgMyc) vectors, with the empty lentiCRISPR v2 vector as a negative control (sgCon). To generate FTO/Myc‐knockout cells, OCI‐Ly1 cells were transfected with sgFTO, sgMyc, or sgCon vectors using the polyethylenimine transfection reagent. After transfection, puromycin (1 μg/mL) was added to select positive clones for 3 days. The sgRNA oligonucleotide sequences are provided in Table S1.

### 2.4 Reverse Transcription Quantitative Polymerase Chain Reaction

Total RNA was extracted from incubated cells using the MolPure cell RNA kit (#19231ES50; Yeasen). cDNA was synthesized using the Hifair AdvanceFast 1st strand cDNA synthesis kit (#11149ES60; Yeasen). Quantitative polymerase chain reaction (qPCR) was performed using Hieff UNICON advanced qPCR SYBR master mix (#11185ES08; Yeasen). Glyceraldehyde‐3‐phosphate dehydrogenase (GAPDH) served as an internal control, and relative expression levels were calculated using the 2^−ΔΔCt^ method. The qPCR primer sequences are listed in Table S1.

### 2.5 Western Blot

Total cell lysates were acquired with RIPA buffer (#P0038; Beyotime). Protein samples (20 μg) were first separated using a 10% sodium dodecyl sulfate–polyacrylamide gel electrophoresis gel, followed by electro‐transformation onto a polyvinylidene fluoride membrane. After blocking with 5% nonfat milk, the membranes were then incubated overnight with primary antibodies, including FTO (#AF6936; Beyotime), Myc (#GTX628459; GeneTex, Irvine, CA, USA), and GAPDH (#AF1186; Beyotime). After washing, the membranes were incubated with horseradish peroxidase‐conjugated secondary antibodies (#A0208; Beyotime). Protein bands were detected using a chemiluminescence detection kit (#E422; Vazyme, Nanjing, China).

### 2.6 Cell Counting Kit‐8 Assay

Transfected lymphoma cells (5 × 10^3^ cells/well) were seeded in 96‐well plates and incubated for 0–72 h. For ibrutinib resistance analysis, the transfected lymphoma cells (5 × 10^3^ cells/well) were treated with different concentrations of ibrutinib (0, 1, 2, 4, 8, 16, or 32 nM) for 24 h. Next, the cell counting kit‐8 (CCK‐8) solution (10 μL) (#40203ES76; Yeasen) was added and incubated for another 2 h. Optical density was measured using a microplate reader (Synergy H1; Bio‐Tek, Winooski, VT, USA) at 450 nm. Based on the survival curves, the half‐maximal inhibitory concentration (IC_50_) was calculated.

### 2.7 Colony Formation Assay

Cells (5 × 10^2^ cells/well) were seeded into six‐well plates with or without ibrutinib (2 nM) and cultured for 14 days. After fixing, cell colonies were stained with 1% crystal violet (#C0121; Beyotime) and counted under an inverted optical microscope (Olympus IX73, Tokyo, Japan).

### 2.8 Flow Cytometry Assay

Cell apoptosis was detected using the Annexin V‐FITC/propidium iodide apoptosis detection kit (#A211; Vazyme) according to the manufacturer's instructions. Samples were analyzed on a FACScan flow cytometer (Becton Dickinson, San Jose, CA, USA).

### 2.9 RNA Immunoprecipitation

RNA immunoprecipitation (RIP) was performed using the BersinBio RIP Kit (#Bes5101; BersinBio, Guangzhou, China). Briefly, cell lysates were incubated overnight at 4°C with anti‐FTO (#14692; Cell Signaling Technology, Beverly, MA, USA) or anti‐IgG (#ab6715; Abcam, Cambridge, MA, USA) antibodies. Afterwards, immuno‐precipitates were incubated with lysis buffer containing protein A/G magnetic beads at 4°C. After digestion with proteinase K, immunoprecipitated RNAs were isolated and then analyzed via real‐time qPCR (RT‐qPCR).

### 2.10 Methylated RNA Immunoprecipitation–Quantitative Polymerase Chain Reaction Assay

The MeRIP assay was performed using the commercial Magna MeRIP m6A Kit (Millipore, Shanghai, China). Total RNA was initially fragmented, then incubated with m6A‐specific antibody and protein A/G magnetic beads. After 2 h, immunoprecipitated RNAs were eluted from the beads with elution buffer, and enriched RNAs were quantified via RT‐qPCR.

### 2.11 mRNA Stability Assay

OCI‐Ly1 cells transduced with sh‐NC or FTO‐sh2 were treated with actinomycin D (10 μg/mL; #53600ES08; Yeasen) for 0, 50, 100, and 150 min. Total RNA was extracted, and Myc mRNA abundance was assessed using RT‐qPCR.

### 2.12 Experimental Animals

Female NOD/SCID mice (4 weeks old, 14–16 g) from the Experimental Animal Center of Suzhou University were housed under specific pathogen‐free conditions with access to sterilized food and water ad libitum. All in vivo experiments were conducted in accordance with the Guide for the Care and Use of Laboratory Animals and were approved by the Animal Care Committee of the Affiliated Zhangjiagang Hospital of Soochow University (approval no. KY2024‐007‐01).

### 2.13 Subcutaneous Xenograft Assay

To evaluate the function of FTO, mice were divided into two groups: sgCon and sgFTO. Briefly, OCI‐Ly1 cells carrying sgCon or sgFTO (1 × 10^7^) were injected subcutaneously into the right flank of NOD/SCID mice (*n* = 6 per group). To assess the role of FTO in ibrutinib resistance, mice were assigned to four groups: LV‐Con, LV‐Con + ibrutinib, LV‐FTO, and LV‐FTO + ibrutinib. SU‐DHL‐8 cells carrying LV‐Con or LV‐FTO (1 × 10^7^) were injected subcutaneously into the right flank of NOD/SCID mice. After 2 weeks, mice inoculated with LV‐Con‐transduced SU‐DHL‐8 cells received either ibrutinib (6 mg/kg/day) or vehicle via oral gavage (*n* = 6 per subgroup). Simultaneously, mice inoculated with LV‐FTO‐transduced SU‐DHL‐8 cells received either ibrutinib (6 mg/kg/day) or vehicle via oral gavage (*n* = 6 per subgroup).

To validate the interaction between FTO and Myc in DLBCL progression, mice were divided into three groups: sgCon, sgMyc, and sgMyc + LV‐FTO. OCI‐Ly1 cells carrying sgCon or sgMyc (1 × 10^7^) were injected subcutaneously into the right flank of NOD/SCID mice. After 2 weeks, mice in the sgCon and sgMyc groups were injected intratumorally with lentiviral particles carrying LV‐Con (1.0 × 10^8^ TU/ml, 100 μL), while those in the sgMyc + LV‐FTO group were injected intratumorally with lentiviral particles carrying LV‐FTO. Lentiviral particles were injected daily for 14 consecutive days.

To verify the interaction between FTO and Myc in DLBCL resistance to ibrutinib, mice were assigned to three groups: LV‐Con + ibrutinib, LV‐Myc + ibrutinib, and LV‐Myc + FTO‐sh2 + ibrutinib. NOD/SCID mice were inoculated subcutaneously in the right flank with U2932 cells carrying LV‐Con or LV‐Myc (1 × 10^7^). Adeno‐associated viruses (AAV) expressing sh‐NC (AAV‐sh‐NC) or FTO‐sh2 (AAV‐FTO‐sh2) were constructed. After 2 weeks, mice in the LV‐Con and sgMyc groups were treated with ibrutinib and injected intratumorally with AAV‐sh‐NC, whereas mice in the sgMyc + LV‐FTO group were treated with ibrutinib and injected intratumorally with AAV‐FTO‐sh2 (1.0 × 10^8^ PFU/ml, 100 μL). Viruses were injected daily for 14 consecutive days.

Tumor dimensions (length/width) were recorded every 48 h using precision calipers, and tumor volume was calculated using the formula: volume = (length × width^2^)/2. Following a 4‐week observation period, the mice were euthanized and their tumors were collected.

### 2.14 Statistical Analysis

Statistical analyses were performed using GraphPad Prism 8 (GraphPad Software, San Diego, CA, USA). Quantification data are presented as means ± standard error of the mean (SEM). Data with normal distribution were compared using an unpaired *t*‐test or one/two‐way analysis of variance (ANOVA) with Tukey's multiple comparison test. A *p* < 0.05 was considered statistically significant.

## 3 Results

### 3.1 FTO Contributes to Tumorigenesis in DLBCL

**FIGURE 1:**
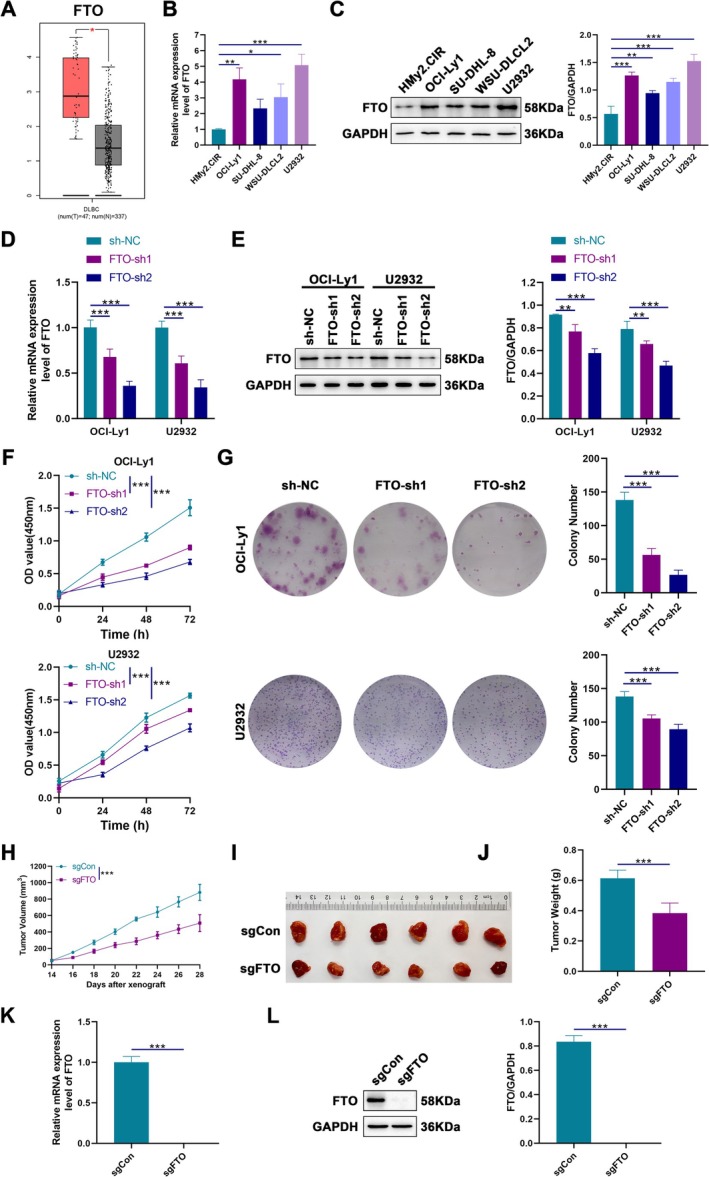
FTO facilitates tumor growth in DLBCL. (A) Data on FTO expression in DLBCL and normal tissues from the GEPIA database. (B) RT‐qPCR analysis of FTO mRNA levels in DLBCL and HMy2.CIR cell lines (*n* = 3). (C) Western blot analysis of FTO protein levels in DLBCL and the HMy2.CIR cell lines (*n* = 3). (D, E) FTO mRNA and protein levels in OCI‐Ly1 and U2932 cells transduced with sh‐NC, FTO‐sh1, or FTO‐sh2, detected via RT‐qPCR or western blot (*n* = 3). (F, G) Cell viability and colony‐forming ability, detected via CCK‐8 and colony formation assays (*n* = 3). (H–J) OCI‐Ly1 cells carrying sgCon or sgFTO were injected subcutaneously into NOD/SCID mice. (H) Tumor size was monitored every 2 days starting on day 14 (*n* = 6). (I, J) At the end of experiments, subcutaneous tumors were dissected, imaged, and weighed (*n* = 6). (K) FTO mRNA levels in subcutaneous tumors detected via RT‐qPCR (*n* = 6). (L) FTO protein levels in subcutaneous tumors detected via western blot (*n* = 3). Error bars stand for mean ± SEM. *p*‐values were calculated using a *t*‐test in (J–L), with one‐way ANOVA in (B, C, G), and two‐way ANOVA in (D, E, F, H). *p < 0.05, **p < 0.01, and ***p < 0.001. ANOVA, analysis of variance; CCK‐8, Cell Counting Kit‐8; DLBCL, diffuse large B‐cell lymphoma; FTO, fat and obesity‐related protein; GEPIA, Gene Expression Profiling Interactive Analysis; NOD/SCID, non‐obese diabetic/severe combined immunodeficient; RT‐qPCR, real‐time quantitative polymerase chain reaction; SEM, standard error of the mean; sgCon/sgFTO, control or FTO‐targeting single‐guide RNA; sh‐NC, negative control short hairpin RNA.

Using the GEPIA platform, we first analyzed differences in FTO expression between the DLBCL and control groups. FTO expression was significantly upregulated in DLBCL samples (Figure 1A). To validate this finding, we measured FTO mRNA levels in DLBCL cell lines; the results showed that FTO mRNA levels were higher in these cell lines, particularly in the OCI‐Ly1 and U2932 cell lines (Figure 1B). A similar pattern was observed for FTO protein levels in DLBCL cell lines (Figure 1C).

Subsequently, loss‐of‐function assays were performed, confirming that FTO mRNA and protein levels were significantly downregulated in OCI‐Ly1 and U2932 cell lines after FTO‐sh1 or FTO‐sh2 transduction (Figure 1D,E). FTO silencing enhanced sensitivity to ibrutinib in these cell lines (Figure S1A,B)‌ and significantly repressed proliferation in both cell lines (Figure 1F,G).

We next generated stable FTO‐knockout OCI‐Ly1 cells using CRISPR‐Cas9 and injected them subcutaneously into NOD‐SCID mice. The results showed that FTO knockout reduced subcutaneous xenograft growth, reflected by smaller tumor volumes and weights (Figure 1H–J). Xenograft tumor derived from the sgFTO cohort showed no detectable FTO mRNA or protein levels (Figure 1K,L). Collectively, these results highlight the tumor‐promoting role of FTO in DLBCL.

### 3.2 FTO Confers Resistance to Ibrutinib in DLBCL

**FIGURE 2:**
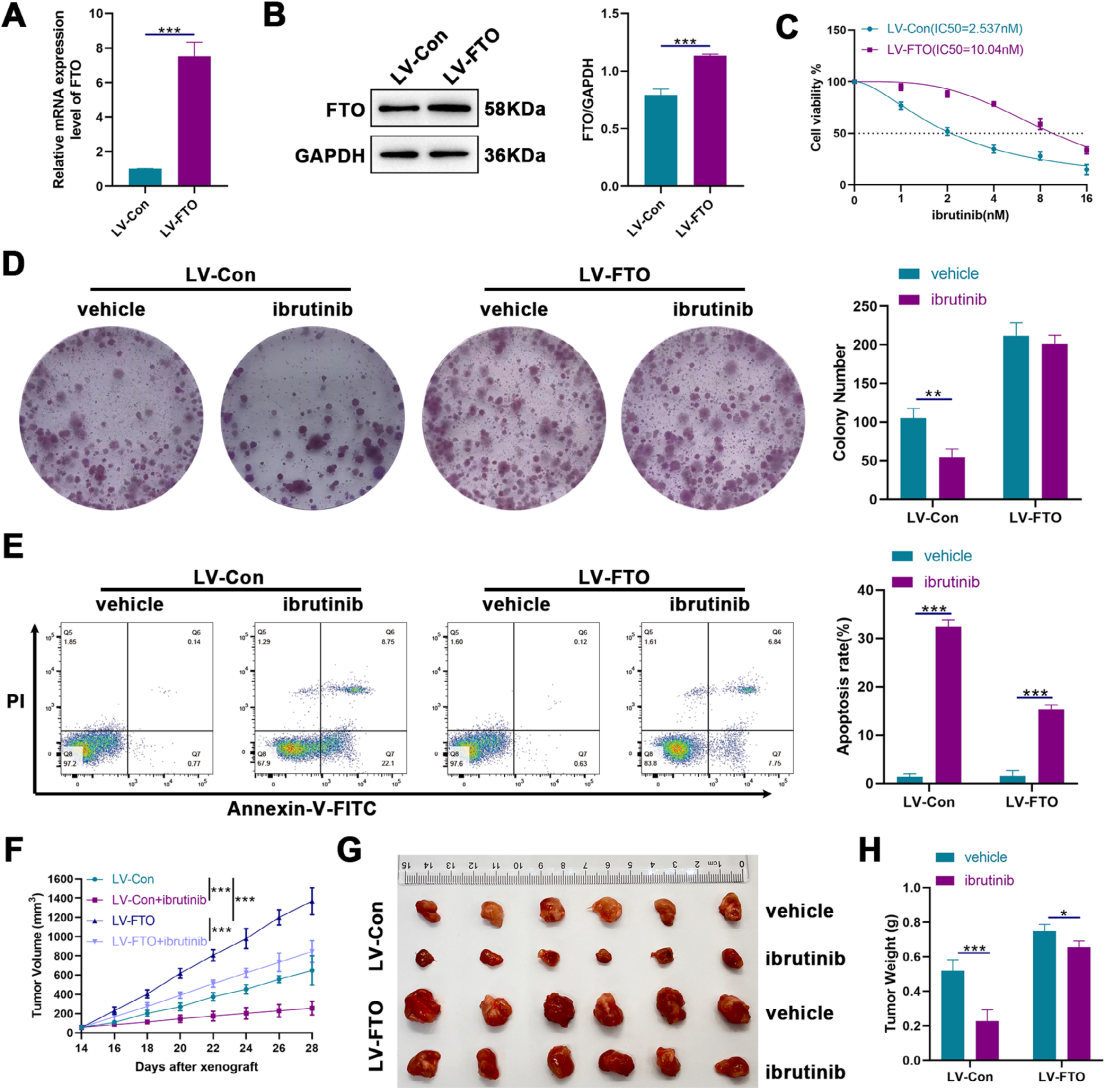
FTO decreases DLBCL sensitivity to ibrutinib. (A, B) FTO mRNA and protein levels in SU‐DHL‐8 cells transduced with LV‐Con or LV‐FTO, detected via RT‐qPCR or western blot (*n* = 3). (C) The IC_50_ value for ibrutinib in SU‐DHL‐8 cells, measured via CCK‐8 assays (*n* = 3). (D, E) The colony formation and apoptosis of SU‐DHL‐8 cells treated with ibrutinib or vehicle were determined by colony formation and flow cytometry assays (*n* = 3). (F–H) SU‐DHL‐8 cells carrying LV‐Con or LV‐FTO were injected subcutaneously into NOD/SCID mice, followed by treatment with ibrutinib or vehicle. (F) Tumor size was measured at the indicated days after injection (*n* = 6). (G) Representative image of xenograft tumors excised from mice within different groups. (H) Tumor weight was analyzed after dissection. Error bars stand for mean ± SEM. *p‐*values were calculated using a *t*‐test in (A, B) and two‐way ANOVA in (C, D, E, F, H). *p < 0.05, **p < 0.01, and ***p < 0.001. ANOVA, analysis of variance; CK‐8, Cell Counting Kit‐8; DLBCL, diffuse large B‐cell lymphoma; FTO, fat and obesity‐related protein; IC_50_, half‐maximal inhibitory concentration; LV‐Con/LV‐FTO, control or FTO‐overexpressing lentiviral vector; RT‐qPCR, real‐time quantitative polymerase chain reaction; SEM, standard error of the mean.

To investigate the effect of FTO upregulation on ibrutinib resistance in DLBCL, we performed gain‐of‐function assays in SU‐DHL‐8 cells, which have relatively low FTO expression, and were transfected with FTO overexpression plasmids. The results showed that FTO mRNA and protein levels were higher in SU‐DHL‐8 cells following LV‐FTO transfection (Figure 2A,B) and that the IC_50_ value for ibrutinib was approximately 3.96 times higher in FTO‐overexpressed cells (Figure 2C). Ibrutinib treatment reduced the colony‐forming capacity of SU‐DHL‐8 cells but not FTO‐overexpressing cells (Figure 2D). Furthermore, ibrutinib induced apoptosis in SU‐DHL‐8 cells with or without FTO overexpression; however, the apoptotic rate remained lower in FTO‐overexpressing cells (Figure 2E).

In subcutaneous xenograft models, mice in the LV‐FTO group had larger and heavier tumors than those in the LV‐Con group. Ibrutinib treatment inhibited subcutaneous xenograft tumor growth in both groups; however, its efficacy was stronger in the LV‐Con group than in the LV‐FTO group (Figure 2F–H). Together, these findings show that FTO confers resistance to ibrutinib in DLBCL.

### 3.3 FTO Mediates m6A‐Demethylation of Myc in DLBCL Cells

**FIGURE 3:**
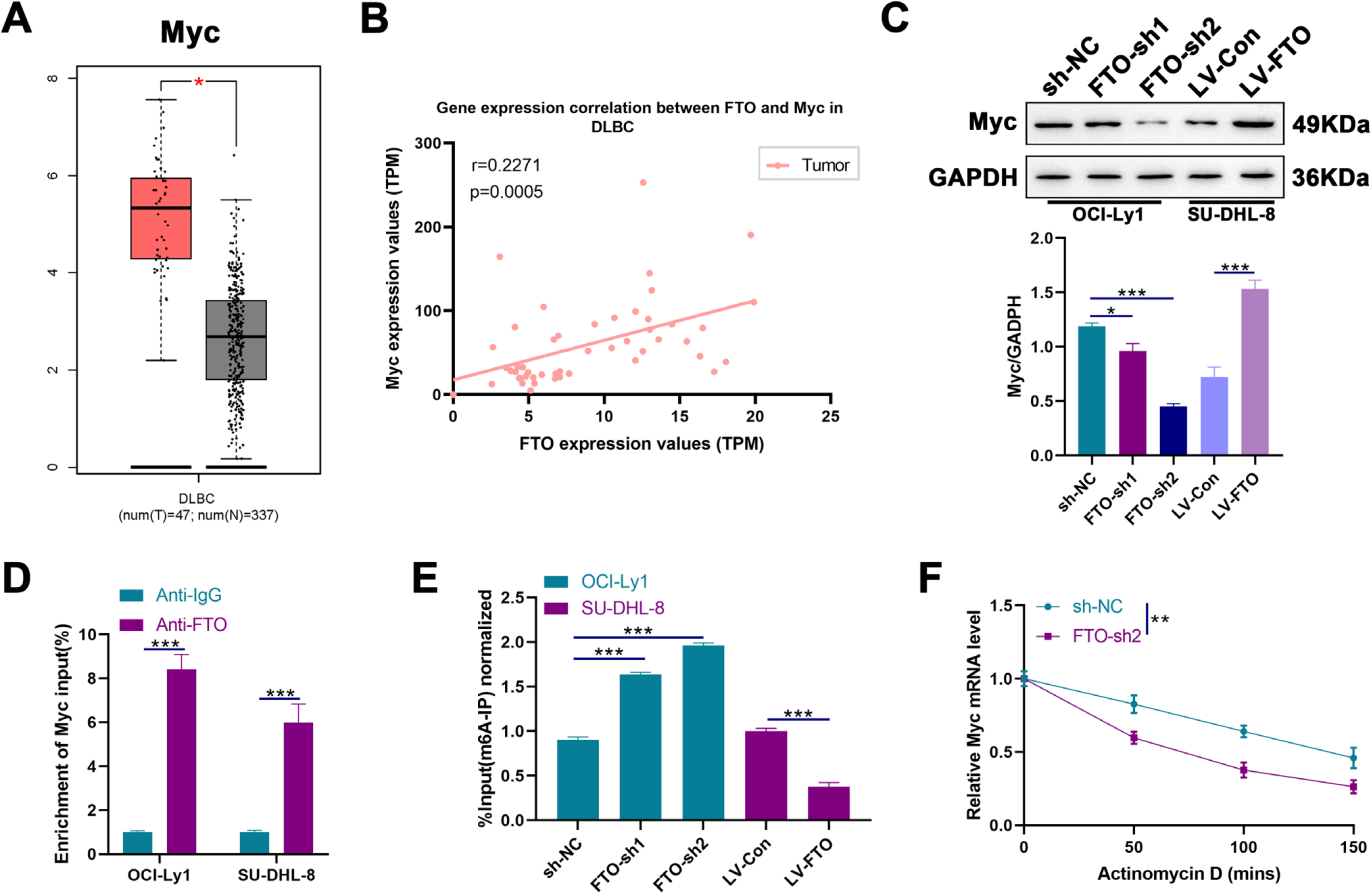
FTO regulates Myc expression via m6A‐demethylation in DLBCL. (A) Myc expression in DLBCL and normal tissues from the GEPIA database. (B) Correlation between FTO and Myc in DLBCL from the MEM database. (C) Protein levels of Myc in FTO‐knockdown OCI‐Ly1 cells and FTO‐overexpressed SU‐DHL‐8 cells, detected via western blot (*n* = 3). (D) RIP analysis of the interaction between FTO and Myc in both OCI‐Ly1 and SU‐DHL‐8 cells with anti‐FTO and anti‐IgG antibodies (*n* = 3). (E) The m6A levels of Myc mRNA in FTO‐knockdown OCI‐Ly1 cells and FTO‐overexpressed SU‐DHL‐8 cells, measured using MeRIP‐qPCR assays (*n* = 3). (F) Relative mRNA expression levels of Myc, analyzed using RT‐qPCR in actinomycin D‐treated OCI‐Ly1 cells (*n* = 3). Error bars stand for mean ± SEM. *p*‐values were calculated using one‐way ANOVA in (C, E) and two‐way ANOVA in (D, F). *p < 0.05, **p < 0.01, and ***p < 0.001. ANOVA, analysis of variance; DLBCL, diffuse large B‐cell lymphoma; FTO, fat and obesity‐related protein; GEPIA, Gene Expression Profiling Interactive Analysis; IgG, immunoglobulin G; m6A, N6‐methyladenosine; MEM, multi‐experiment matrix; MeRIP‐qPCR, methylated RNA immunoprecipitation followed by quantitative polymerase chain reaction; Myc, MYC proto‐oncogene protein; RIP, RNA immunoprecipitation; RT‐qPCR, real‐time quantitative polymerase chain reaction; SEM, standard error of the mean.

Using the GEPIA platform, we confirmed significant upregulation of Myc in DLBCL (Figure 3A). Furthermore, FTO showed a positive correlation with Myc in DLBCL, as predicted by the MEM database (Figure 3B). Myc protein levels were lower in FTO‐knockdown OCI‐Ly1 cells and higher in FTO‐overexpressing SU‐DHT‐8 cells (Figure 3C). RIP assays indicated that Myc could be enriched by an anti‐FTO antibody, suggesting FTO–Myc binding (Figure 3D).

Notably, FTO silencing elevated the m6A levels of Myc mRNA, whereas FTO overexpression reduced them (Figure 3E). Moreover, FTO inhibition decreased Myc mRNA levels in OCI‐Ly1 cells treated with actinomycin D, suggesting that FTO silencing reduced the stability of Myc mRNA (Figure 3F). Altogether, these results suggest that FTO regulates Myc expression through m6A‐dependent demethylation in DLBCL.

### 3.4 FTO Promotes DLBCL Progression Partly Through a Myc‐Dependent Mechanism

**FIGURE 4:**
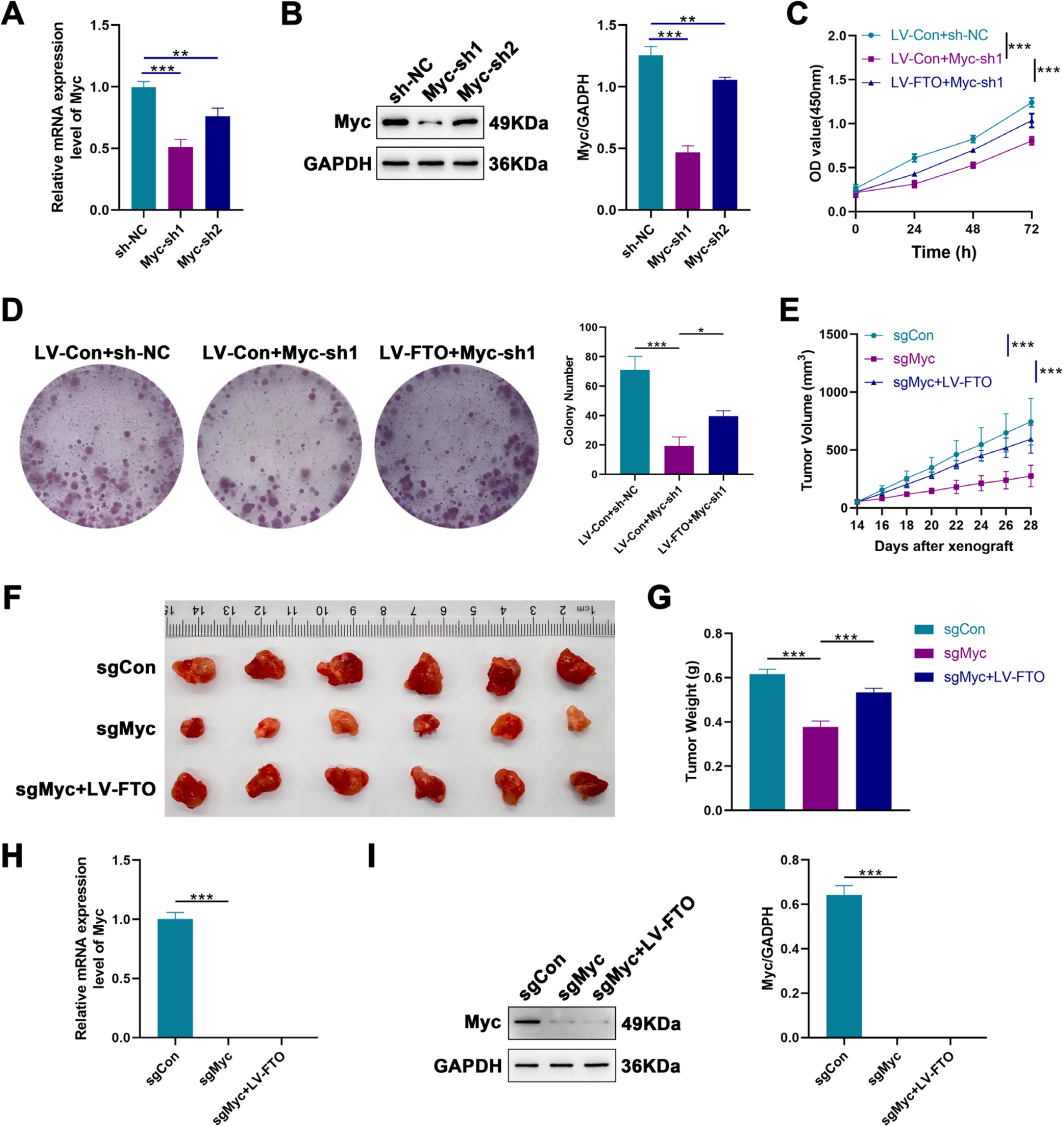
FTO promotes tumor growth in DLBCL by elevating Myc expression. (A, B) Myc mRNA and protein levels in SU‐DHT‐8 cells transduced with sh‐NC, Myc‐sh1, or Myc‐sh2, detected via RT‐qPCR or western blot (*n* = 3). (C, D) The viability and colony formation of SU‐DHT‐8 cells transduced with LV‐Con + sh‐NC, LV‐Con + Myc‐sh1, or LV‐FTO + Myc‐sh1, evaluated using CCK‐8 and colony formation assays (*n* = 3). (E–G) NOD/SCID mice were inoculated subcutaneously with OCI‐Ly1 cells carrying sgCon or sgMyc, followed by intra‐tumoral injection with lentiviral particles carrying LV‐Con or LV‐FTO. (E) The growth curves of xenograft tumors within mice in different groups (*n* = 6). (F, G) Representative images and tumor weight of xenograft tumors in different groups (*n* = 6). (H, I) The mRNA and protein levels of Myc in xenograft tumors, measured using RT‐qPCR (*n* = 6) and western blot (*n* = 3). Error bars stand for mean ± SEM. *p*‐values were calculated using one‐way ANOVA in (A, B, D, G–I) and two‐way ANOVA in (C, E). *p < 0.05, **p < 0.01, and ***p < 0.001. ANOVA, analysis of variance; CCK‐8, Cell Counting Kit‐8; DLBCL, diffuse large B‐cell lymphoma; FTO, fat and obesity‐related protein; LV‐Con/LV‐FTO, control or FTO‐overexpressing lentiviral vector; Myc, MYC proto‐oncogene protein; Myc‐sh1/Myc‐sh2, Myc‐targeting shRNAs; RT‐qPCR, real‐time quantitative polymerase chain reaction; SEM, standard error of the mean; sgCon/sgMyc, control or Myc‐targeting single‐guide RNA; sh‐NC, negative control short hairpin RNA.

Rescue experiments were performed to assess whether FTO modulates DLBCL progression via interaction with Myc. Myc interference was achieved in SU‐DHL‐8 cells by transducing Myc‐sh1 or Myc‐sh2; Myc‐sh1, with a greater interference efficiency, was chosen for subsequent exploration (Figure 4A,B). Myc knockdown reduced SU‐DHL‐8 cell proliferation. However, the promoting effect of FTO up‐regulation on SU‐DHL‐8 cell proliferation was attenuated following co‐transfection with Myc‐sh1 (Figure 4C,D).

Subcutaneous xenograft assays showed that mice within the sgMyc group had smaller tumor volumes and lighter tumor weights than those within the sgNC group did. However, intra‐tumoral injection of lentiviral particles carrying LV‐FTO partially counteracted the inhibitory effect of sgMyc on tumor growth (Figure 4E–G), suggesting that FTO may also act through Myc‐independent pathways. We further detected Myc mRNA and protein levels in tumor samples and confirmed Myc knockdown (Figure 4H,I). These results show that DLBCL progression is driven by FTO, at least in part via a Myc‐dependent mechanism.

### 3.5 FTO Promotes Ibrutinib Resistance in DLBCL Partly Through Myc‐Dependent Mechanisms

**FIGURE 5:**
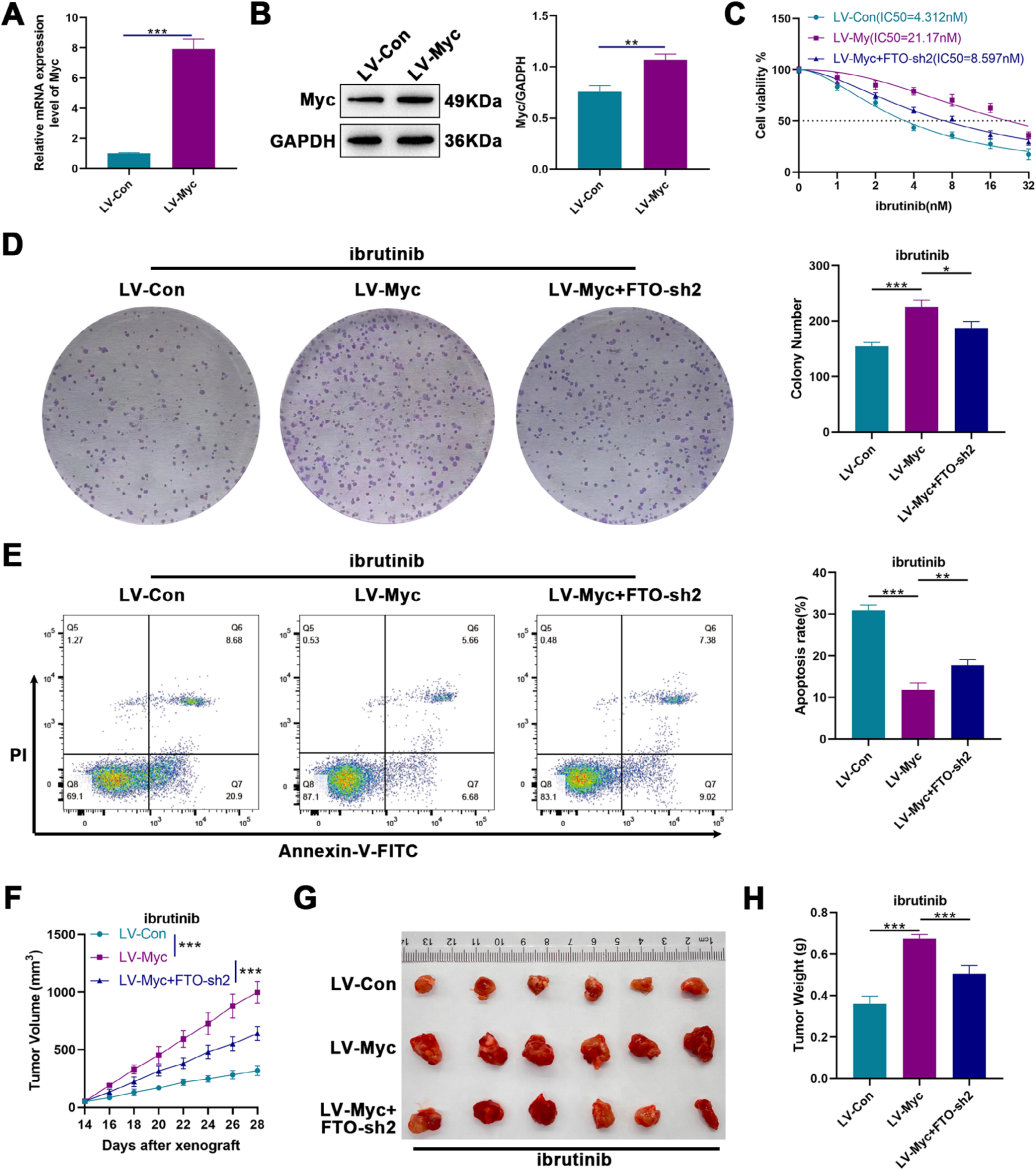
FTO decreases ibrutinib sensitivity in DLBCL by mediating Myc expression. (A, B) Analysis of Myc mRNA and protein levels in U2932 cells transduced with LV‐Con or LV‐Myc, detected via RT‐qPCR or western blot (*n* = 3). (C) The IC_50_ value for ibrutinib in U2932 cells transduced with LV‐Con, LV‐Myc, or LV‐Myc + FTO‐sh2, measured via CCK‐8 assays (*n* = 3). (D, E) The colony formation and apoptosis of U2932 cells under ibrutinib or vehicle treatment, determined using colony formation and flow cytometry assays (*n* = 3). (F–H) NOD/SCID mice were inoculated subcutaneously with U2932 cells carrying LV‐Con or LV‐Myc, followed by intra‐tumoral injection with AAV‐sh‐NC or AAV‐FTO‐sh2. (F) The growth curves of xenograft tumors (*n* = 6). (G) Representative image of xenograft tumors excised from mice. (H) Tumor weight of xenograft tumors. Error bars stand for mean ± SEM. *p*‐values were calculated using a *t*‐test in (A, B), one‐way ANOVA in (D, E, H), and two‐way ANOVA in (C, F). *p < 0.05, **p < 0.01, and ***p < 0.001. AAV‐sh‐NC/AAV‐FTO‐sh2, adeno‐associated virus carrying control or FTO‐targeting shRNA; ANOVA, analysis of variance; CCK‐8, Cell Counting Kit‐8; DLBCL, diffuse large B‐cell lymphoma; FTO, fat and obesity‐related protein; FTO‐sh2, FTO‐targeting shRNA; IC_50_, half‐maximal inhibitory concentration; LV‐Con/LV‐Myc, control or Myc‐overexpressing lentiviral vector; Myc, MYC proto‐oncogene protein; RT‐qPCR, real‐time quantitative polymerase chain reaction; SEM, standard error of the mean.

We next examined whether FTO modulates ibrutinib resistance in DLBCL by interacting with Myc. To validate the function of Myc, U2932 cells with stable Myc overexpression were generated, which showed markedly increased Myc mRNA and protein levels following LV‐Myc transduction (Figure 5A,B). Myc overexpression increased the IC_50_ value for ibrutinib, but this effect was attenuated following FTO knockdown (Figure 5C). Myc overexpression also enhanced cell colony formation and suppressed cell apoptosis in U2932 cells under ibrutinib treatment, whereas FTO knockdown partially reversed these changes (Figure 5D,E).

Consistent with the in vitro findings, Myc overexpression promoted tumor growth in ibrutinib‐treated xenograft models, while intra‐tumoral injection of AAV‐FTO‐sh2 attenuated this tumor‐promoting effect (Figure 5F–H). Collectively, these results show that FTO enhances cell resistance to ibrutinib in DLBCL in a partially Myc‐dependent manner.

## 4 Discussion

FTO, a key m6A demethylase, has been widely implicated in tumor progression and drug resistance. However, its role in ibrutinib resistance in DLBCL remains incompletely elucidated. Prior research has shown that FTO mediates chemoresistance in leukemia and solid tumors. For example, Wang et al. reported that FTO upregulates SOD2 expression, thereby promoting bortezomib resistance in multiple myeloma [21], and Lu et al. showed that FTO‐mediated demethylation of LINC01559 promotes docetaxel resistance in breast cancer [22]. Conversely, FTO can also sensitize tumors to therapy, as shown in ovarian cancer, where it activates NLRP3 inflammasome‐mediated pyroptosis, thereby increasing the sensitivity of ovarian cancer to cisplatin [23]. Specifically, higher FTO levels have been reported in DLBCL cell lines (RCK‐8, LY‐3, DHL‐6, and U2932), and FTO silencing in U2932 cells has been shown to inhibit cell activity and promote apoptosis, whereas FTO‐overexpressing LY‐3 cells showed the opposite results [12].

Consistent with these findings, we confirmed FTO upregulation in DLBCL cells. FTO silencing reduced OCI‐Ly1 and U2932 cell viability and colony‐forming capacity, whereas FTO overexpression elevated ibrutinib IC_50_ values and decreased ibrutinib‐mediated proliferation repression and apoptosis induction in SU‐DHL‐8 cells. In vivo, FTO knockout with CRISPR‐Cas9 decreased tumor growth in subcutaneous xenograft models, whereas FTO overexpression enhanced tumor growth and ibrutinib resistance. Collectively, these findings establish that FTO promotes tumor growth and reduces sensitivity to ibrutinib in DLBCL.

Myc overexpression is common in DLBCL and has been associated with poor survival [24]. Moyo et al. reported that high Myc expression activates the BCR pathway and increases ibrutinib resistance in precancerous B cells [25]. FTO has also been linked to Myc regulation across multiple cancers; it stabilizes Myc mRNA through m6A methylation and promotes tumor progression in gastric [19], cervical [26], and colorectal cancers [20]; its inhibition elevates temozolomide sensitivity in glioma by targeting Myc [18]. In addition, FTO‐mediated m6A‐demethylation of Myc decreases gefitinib sensitivity in lung cancer [27].

Consistent with these findings, we demonstrated that FTO stabilizes Myc mRNA by regulating m6A demethylation of Myc in DLBCL cells. Myc knockdown decreased SU‐DHL‐8 cell proliferation and attenuated the promoting effect of FTO on SU‐DHL‐8 cell proliferation. In contrast, Myc overexpression elevated the IC_50_ value for ibrutinib, promoted cell colony formation, and inhibited cell apoptosis in SU‐DHL‐8 cells under ibrutinib treatment, whereas FTO silencing reversed these Myc‐induced changes. Similarly, Myc knockout with CRISPR‐Cas9 suppressed tumor growth in vivo, but intra‐tumoral FTO delivery weakened this suppressive effect. Conversely, Myc overexpression promoted tumor growth under ibrutinib treatment, whereas intra‐tumoral FTO knockdown attenuated this response. These findings show that FTO promotes DLBCL progression, in part, by stabilizing Myc, although additional Myc‐independent mechanisms are likely involved and warrant further investigation. For example, FTO‐mediated m6A demethylation of SIVA1, NEDD4, and NUPR1 contributes to tumor progression in other cancers [7, 28, 29], suggesting broader multi‐pathway regulation; however, whether FTO participates in DLBCL progression through these mechanisms remains unknown.

Beyond the demethylase route exemplified by FTO, other m6A regulators can also converge on Myc and influence drug response. The m6A writer METTL3 is upregulated in colorectal cancer and promotes tumor progression at least in part by enhancing MYC expression in an m6A‐IGF2BP1‐dependent manner [30]. Among m6A readers, YTHDF1 is oncogenic in colorectal cancer, linked to c‐Myc signaling, and its inhibition re‐sensitizes chemoresistant colon cancer cells [31]. In lymphoma, METTL3‐mediated m6A writing and YTHDF2 recognition regulate C1qA and modulate rituximab resistance in DLBCL, underscoring that multiple nodes of the m6A machinery shape therapeutic response in this disease [11]. Collectively, these comparisons position our findings as a demethylase‐centered, DLBCL‐ and ibrutinib‐specific mechanism, complementary to writer/reader routes that can also affect MYC and drug resistance.

FTO‐mediated m6A demethylation of Myc has been described in several solid tumors; nonetheless, our findings expand this regulatory paradigm to DLBCL and, importantly, to ibrutinib resistance, an association that has not been previously reported. DLBCL differs from solid tumors in its dependence on BCR and BTK signaling, where therapeutic resistance commonly results from adaptive transcriptional reprogramming rather than fixed genetic alterations. Therefore, showing that FTO sustains Myc expression via m6A demethylation to maintain BTK‐driven survival signaling provides novel insight into the epitranscriptomic mechanisms underlying ibrutinib resistance in lymphoma.

This study has some limitations. We were unable to collect DLBCL‐related clinical samples to validate the findings. Beyond Myc, FTO may influence additional m6A‐modified transcripts, collectively shaping lymphoma biology. For instance, FTO‐mediated demethylation of FLOT2 has been reported to enhance DLBCL aggressiveness through PI3K/AKT/mTOR signaling [12]. In acute myeloid leukemia, FTO reduces m6A levels on ASB2 and RARA, thereby restraining differentiation and shaping drug response [32]. In melanoma, FTO stabilizes PD‐1, CXCR4, and SOX10, reducing sensitivity to anti‐programmed death‐1 therapy [33]. Given FTO's broad substrate specificity, additional targets involved in apoptosis, metabolism, or cell signaling likely contribute to its effects. Future studies integrating RNA‐seq and m6A‐seq analyses will be valuable to determine whether MYC is the predominant functional effector or one of several cooperative downstream pathways.

In conclusion, our findings show that FTO promotes DLBCL growth and ibrutinib resistance primarily through rm6A‐dependent stabilization of Myc, highlighting FTO's potential as a therapeutic target for overcoming ibrutinib resistance in DLBCL.

## Conflicts of Interest

The authors declare no conflicts of interest.

## Supporting information


**FIGURE S1:** Effect of FTO knockdown on ibrutinib resistance in OCI‐Ly1 and U2932 cells. (A, B) The IC_50_ value for ibrutinib in FTO‐knockdown OCI‐Ly1 and U2932 cells, measured via CCK‐8 assays (*n* = 3). Error bars stand for mean ± SEM. FTO, fat and obesity‐related protein; CCK‐8, Cell Counting Kit‐8; SEM, standard error of the mean; IC_50_, half‐maximal inhibitory concentration.

**TABLE S1:** The oligonucleotide sequences used in the study.

## Data Availability

The data that support the findings of this study are available from the corresponding author upon reasonable request.
