# Supplementary figures and images for: Fat Mass and Obesity‐Associated Protein Contributes to Tumorigenesis and Drug Resistance of Diffuse Large B‐Cell Lymphoma by Suppressing N6‐Methyladenosine Methylation of Myc
Source: Kaohsiung J Med Sci. 2026 Jan 13;42(7):e70158. doi: 10.1002/kjm2.70158 (PMC13344435; doi:10.1002/kjm2.70158)

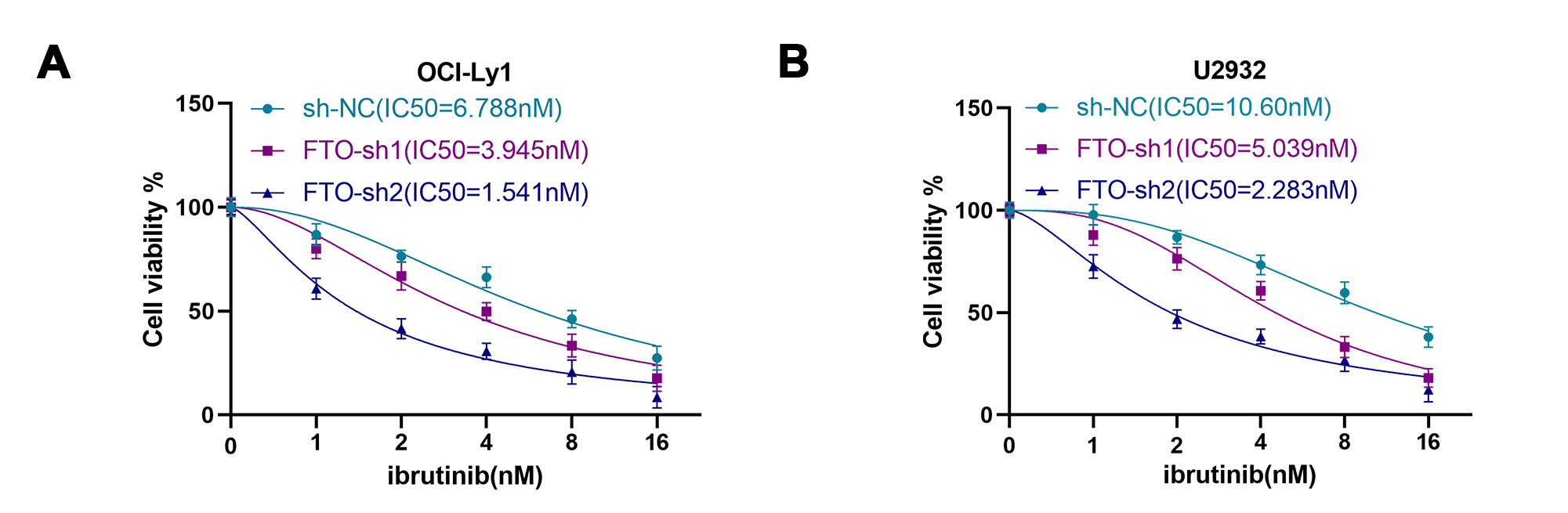

Supplement: Supplementary file 1 — FIGURE S1: Effect of FTO knockdown on ibrutinib resistance in OCI‐Ly1 and U2932 cells. (A, B) The IC50 value for ibrutinib in FTO‐knockdown OCI‐Ly1 and U2932 cells, measured via CCK‐8 assays (n = 3). Error bars stand for mean ± SEM. FTO, fat and obesity‐related protein; CCK‐8, Cell Counting Kit‐8; SEM, standard error of the mean; IC50, half‐maximal inhibitory concentration. [file KJM2-42-e70158-s002.tif]
