# Supplementary material for: Fat Mass and Obesity‐Associated Protein Contributes to Tumorigenesis and Drug Resistance of Diffuse Large B‐Cell Lymphoma by Suppressing N6‐Methyladenosine Methylation of Myc
Source: Kaohsiung J Med Sci. 2026 Jan 13;42(7):e70158. doi: 10.1002/kjm2.70158 (PMC13344435; doi:10.1002/kjm2.70158)
Supplement: Supplementary file 2 — TABLE S1: The oligonucleotide sequences used in the study. [file KJM2-42-e70158-s001.docx]

**Supplementary table 1 The oligonucleotide sequences used in the study**

| **Oligonucleotides** |
| --- |
| Oligonucleotides for FTO-sh1 forward:  5’-CCGGCAACGTAACTTTGCTGAATTTCTCGAGAAATTCAGCAAAGTTACGTTGTTTTTG-3’ |
| Oligonucleotides for FTO-sh1 reverse:  5’-AATTCAAAAACAACGTAACTTTGCTGAATTTCTCGAGAAATTCAGCAAAGTTACGTTG-3’ |
| Oligonucleotides for FTO-sh2 forward:  5’-CCGGGCCAGTGAAAGGGTCTAATATCTCGAGATATTAGACCCTTTCACTGGCTTTTTG-3’ |
| Oligonucleotides for FTO-sh2 reverse:  5’-AATTCAAAAAGCCAGTGAAAGGGTCTAATATCTCGAGATATTAGACCCTTTCACTGGC-3’  Oligonucleotides for Myc-sh1 forward:  5’-CCGGCCTGTGCCACTAAACTACATTCTCGAGAATGTAGTTTAGTGGCACAGG TTTTTG-3’  Oligonucleotides for Myc-sh1 reverse:  5’-AATTCAAAAACCTGTGCCACTAAACTACATTCTCGAGAATGTAGTTTAGTGGCACAGG-3’  Oligonucleotides for Myc-sh2 forward:  5’-CCGGCATTGGCTCTTCTCAAGCTCT CTCGAGAGAGCTTGAGAAGAGCCAATG TTTTTG-3’  Oligonucleotides for Myc-sh2 reverse:  5’-AATTCAAAAACATTGGCTCTTCTCAAGCTCTCTCGAGAGAGCTTGAGAAGAGCCAATG -3’  Oligonucleotides for sgFTO forward:  5’-CACCGGACCTGCACCATGGTCCGAG-3’  Oligonucleotides for sgFTO reverse:  5’-AAACCTCGGACCATGGTGCAGGTCC-3’  Oligonucleotides for sgMyc forward:  5’-CACCGGACGCGGGGAGGAAATTGAC-3’  Oligonucleotides for sgMyc reverse:  5’-AAACGTCAATTTCCTCCCCGCGTCC-3’ |
| qPCR primers for FTO forward: 5’-CCAGAACCTGAGGAGAGAATGG-3’  qPCR primers for FTO reverse: 5’-CGATGTCTGTGAGGTCAAACGG-3’  qPCR primers for Myc reverse: 5’-CCTGGTGCTCCATGAGGAGAC-3’  qPCR primers for Myc reverse: 5’-CAGACTCTGACCTTTTGCCAGG-3’  qPCR primers for GAPDH reverse: 5’-GTCTCCTCTGACTTCAACAGCG-3’  qPCR primers for GAPDH reverse: 5’-ACCACCCTGTTGCTGTAGCCAA-3’ |
